# Indirect markers of fibrosis in chronic liver diseases: Is aspartate transaminase-to-platelet ratio (APRI) a useful test?

**Published:** 2011-07-01

**Authors:** Claudia Della Corte, Anna Alisi, Valerio Nobili

**Affiliations:** 1 Unit of Liver Research of Bambino Gesu Children's Hospital, Rome, Italy

**Keywords:** Fibrosis, Liver diseases, NAFLD

Dear Editor,

We read with great interest the article entitled "Noninvasive assessment of liver fibrosis with the aspartate transaminase to platelet ratio (APRI): Usefulness in patients with chronic liver disease" by Yilmaz Y et al. published in a recent issue of Hepatitis Monthly[1]. In this study, the authors investigated the diagnostic power of APRI for predicting hepatic fibrosis in patients with chronic hepatitis B (CHB) and C (CHC) and non-alcoholic fatty liver disease (NAFLD). The study was retrospectively conducted on adults with chronic liver disease (CHB, CHC, or NAFLD) at the Department of Gastroenterology of Marmara University (Istanbul). Yilmaz et al. demonstrated that APRI was significantly associated with fibrosis scores in patients with CHC and NAFLD, but not in those with CHB. Interestingly, the analysis of the ROC curves highlighted that APRI was able to provide an acceptable prediction of the presence of liver fibrosis in patients with CHC (0.582, P < 0.01) and NAFLD (0.627, P < 0.01), but not in those with CHB (0.541, P = NS). Therefore, the authors concluded that APRI might be a useful test for the detection of liver fibrosis which develops in specific etiologies, including NAFLD and hepatitis virus C infection[1]. APRI is one of the many indirect, non-invasive markers of hepatic fibrosis developed in recent years to avoid the use of liver biopsy, which, although still considered the gold standard for evaluation of fibrosis, is limited by its invasiveness, risk of complications, sampling errors, and variability in pathological interpretation. APRI is composed of panels that are routinely performed in patients with liver disease, and therefore, could be a practical and convenient way to follow patients. However, as demonstrated by Yilmaz et al., indirect markers such as APRI should be used with caution because their sensitivity in predicting fibrosis can be strongly influenced by the etiology of fibrosis. A relevant limitation of this study is that it was conducted in a tertiary liver centre, where the higher proportion of patients with advanced diseases might represent a selection bias with respect to a population observed in a more general setting. An additional limitation of APRI is represented by its inability to identify mild forms of liver fibrosis. In fact, aspartate transaminase (AST) serum levels and platelet counts become altered mainly in advanced stages of hepatic disease; thus, APRI could be ineffective in detecting early liver fibrosis.

**Figure 1:**
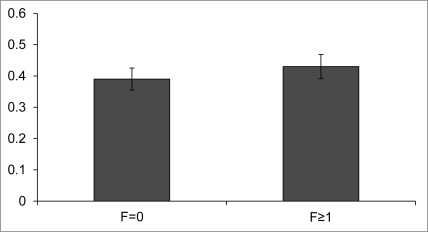
Histogram represents the median value of APRI ± SD in 100 children with biopsy-proven NAFLD separated on the basis of presence of fibrosis: 35 patients with F = 0 and 65 patients with F ≥ 1.

Furthermore, we would like to highlight the fact that at least one other factor has to be carefully considered in the use of indirect markers such as APRI: the age of patients. In fact, some pediatric studies demonstrated that APRI was diagnostically useful only in a specific pediatric setting[2][3][4]. Moreover, although Yilmaz et al. had shown that APRI is a useful marker for liver fibrosis in adult patients with NAFLD, this is not the case in pediatric patients with the same disease. In fact, as shown in Figure 1, we found no statistically significant difference between the APRI value from NAFLD children without fibrosis (F = 0) and those with fibrosis (F ≥ 1). This analysis was retrospectively conducted on 100 children with biopsy-proven NAFLD observed at our tertiary centre of Liver Unit of the Bambino Gesu Children Hospital. Interestingly, a recent study conducted on the same patients demonstrated that serum levels of hyaluronic acid (HA) are good predictors of the degree of hepatic fibrosis, independent of others biochemical indices of liver damage[5].

In our opinion, this finding makes APRI not suitable as a screening program and not adequate to substitute liver biopsy, but it could be used to correctly identify patients who should be evaluated by biopsy because of the high probability of detecting advanced liver damage. Noteworthy, the ideal non-invasive method of assessment of liver fibrosis would be available at any level of health care, from general practice to tertiary care and would have the same diagnostic power both in adults and in children, independent of the severity of the disease.

In conclusion, although we believe that greater emphasis and importance should be given to the research on novel non-invasive fibrosis markers, more studies are needed to identify an optimal prediction system useful both in adults and in children.
